# Adipose tissue-specific *Nrf2* knockdown inhibits the cGAS-STING pathway to attenuate inflammation in obese mice

**DOI:** 10.3389/fendo.2025.1711793

**Published:** 2026-01-12

**Authors:** Lin Zhao, Lina Tuerxunaili, Guiyun Shi, Mengyue Wu, Yi Jiao

**Affiliations:** State Key Laboratory of Pathogenesis, Prevention and Treatment of High Incidence Diseases in Central Asia, Xinjiang Key Laboratory of Molecular Biology for Endemic Diseases, Department of Biochemistry and Molecular Biology, School of Basic Medical Sciences, Xinjiang Medical University, Urumqi, China

**Keywords:** adipose tissue, cGAS-STING, high-fat diet, inflammation, Nrf2, obesity, oxidative stress

## Abstract

**Background:**

Obesity is a chronic, non-infectious inflammatory disease associated with oxidative stress and is triggered by adipose tissue expansion. Nuclear factor erythroid 2-related factor 2 (NRF2) is a core antioxidant defense system transcription factor in adipose tissue and is associated with the cyclic GMP-AMP synthase-stimulator of interferon genes (cGAS-STING) pathway. However, the regulatory roles of NRF2 and the cGAS-STING pathway in obesity-related metabolic disorders remain unclear. Therefore, this study aimed to evaluate the effects of adipose tissue-specific Nrf2 knockout (*Nrf2*^△/adipo^) on obesity-related metabolic phenotypes and inflammation in mice.

**Methods:**

An *Nrf2*^△/adipo^ mouse model was constructed, followed by high-fat diet (HFD) intervention to induce obesity. Additionally, various tests, including glucose tolerance test, RNA sequencing, and western blotting, were performed to elucidate the mechanism of Nrf2 in obesity in the mice.

**Results:**

*Nrf2*^Δ/adipo^ mice exhibited reduced body weight, body fat, triglyceride levels, and total cholesterol content and improved glucose and insulin tolerance compared with HFD *Nrf2*^flox/flox^ controls. *Nrf2*^Δ/adipo^ mice showed decreased body weight and significant increases in oxygen and carbon dioxide consumption and energy expenditure. Transcriptome sequencing and pathway enrichment analysis revealed that adipose tissue-specific *Nrf2* knockdown significantly downregulated cGAS-STING pathway-related genes and their corresponding proteins. Further mechanistic analysis revealed increased mitochondrial DNA copy number in adipose tissue after *Nrf2* knockdown, along with decreased cGAMP and malondialdehyde levels. Flow cytometry analysis revealed a reduction in M1 macrophages following adipose tissue-specific knockout of Nrf2. Enzyme-linked immunosorbent assay revealed decreased expression of proinflammatory factors.

**Conclusion:**

Specific *Nrf2* knockdown in adipose tissue attenuates obesity-induced adipose tissue inflammation by inhibiting the cGAS-STING pathway, providing a novel therapeutic strategy targeting the adipose-specific antioxidant-inflammatory regulatory network.

## 1 Introduction

Obesity is a chronic metabolic disease marked by excessive and often disproportionate fat accumulation, and has become one of the most serious global public health concerns (1, 2). During obesity, adipose tissue more rapidly than its supporting vasculature, resulting in local hypoxia and oxidative stress (3). These conditions damages cells and promote lipid peroxidation of biological membranes. Lipid peroxidation compromises the structural integrity of mitochondrial membranes, allowing mitochondrial DNA (mtDNA) to leak into the cytosol or circulation and trigger an inflammatory response (4). Obesity-induced chronic inflammation is a core pathological process underlying obesity and related metabolic disorders, including type 2 diabetes and cardiovascular disease (5).

Nuclear factor erythroid 2-related factor 2 (NRF2) is a key transcription factor that regulates antioxidant responses (6, 7). Under oxidative stress, Nrf2 translocates to the nucleus and induces the expression of antioxidant enzymes, thereby reducing oxidative damage (8, 9). Beyond its antioxidant function, NRF2 also plays an important role in inflammation and metabolic disease processes (10–12). The cyclic GMP-AMP synthase-stimulator of interferon genes (cGAS-STING) pathway is a major effector in the cellular sensing of double-stranded DNA abnormally present in the cytoplasm, which is mediated by the activation of type I interferon and inflammatory factor expression to facilitate the natural immune response (13). In obesity, persistent oxidative stress induces mitochondrial membrane lipid peroxidation, subsequently compromising membrane integrity and leading to mtDNA leakage into the cytoplasm. This activates the cGAS-STING pathway, thereby triggering an inflammatory response (14).

Although the cGAS–STING pathway is recognized as a critical link between cellular stress and immune activation with its activity potentially regulated by upstream antioxidant signalling. However, it remains unclear whether Nrf2, as a core antioxidant regulator, influences obesity-induced inflammation and metabolic disorders in adipose tissue by modulating the cGAS–STING pathway. To address this gap, we used an adipose tissue-specific Nrf2 knockout mouse model to investigate the tissue-specific role of Nrf2 in obesity-related metabolic disorders. We packed particular emphasis on determining whether Nrf2 participates in these pathological processes by modulating the cGAS–STING pathway.

## 2 Materials and methods

### 2.1 Animals

Adipose tissue-specific *Nrf2* knockout (*Nrf2^Δ^*^/adipo^) mice were generated by breeding *Nrf2*^flox/flox^ (with LoxP sites flanking exon 4-5) with transgenic mice expressing Cre recombinase under the adiponectin promoter (Adipoq-Cre) (Saiye Biotech, Jiangsu, China). Mice were housed in a specific pathogen-free-grade animal house at the Laboratory Animal Center of Xinjiang Medical University, maintained under controlled conditions (temperature, 22 ± 3 °C; humidity, 50 ± 5%; and 12-h light/dark cycle), with *ad libitum* access to food and water. Genomic DNA extracted from clipped mouse toes was used for polymerase chain reaction (PCR). **Supplementary Table S1** lists the primer sequences used for genotyping. Forty male *Nrf2^flox/flox^* and *Nrf2^Δ/adipo^* mice (10–12-week-old) were randomly assigned to normal diet (ND) or high-fat diet (HFD) groups. *Nrf2^flox/flox^* and *Nrf2^Δ/adipo^* mice in the ND group were fed a regular chow diet (ND, HuanYu Bio, 10% fat, Beijing, China) for 21 weeks. *Nrf2^flox/flox^* and *Nrf2^Δ/adipo^* mice in the HFD group were fed high-fat chow (HFD, Research Diet, 60% fat, New Brunswick, NJ, USA) for 21 weeks. Body weight, food intake, water intake, and blood glucose levels were monitored regularly. All animal experiments were approved by the Ethical Review Committee of Xinjiang Medical University (ethical approval number: IACUC-20220310-06). Animal suffering and the number of animals used were minimized.

### 2.2 Intraperitoneal glucose tolerance test

Mice were fasted overnight for 12 h prior to intraperitoneal injection of glucose (1 g/kg body weight). Blood glucose was measured from the tail vein, at 0, 15, 30, 60, 90, and 120 minutes using a blood glucose meter (Roche Diabetes Care GmbH, Mannheim, Germany).

### 2.3 Intraperitoneal insulin tolerance test

Mice were intraperitoneally injected with insulin (1 U/kg body weight) after 5 h of fasting. Venous blood was collected via the tail vein, and blood glucose concentrations were measured at 0, 15, 30, 60, 90, and 120 minutes using a blood glucose meter (Roche Diabetes Care GmbH, Mannheim, Germany).

### 2.4 Measurement of body composition

Body composition(Lean mass, Fat mass, Fluid mass) were assessed using a Minispec LF90II live component analyser (Bruker, Billerica, MA, USA), and body weight was recorded before measurement.

### 2.5 Metabolic cage studies

The mice were housed individually in metabolic cages, maintained at 22 ± 3 °C under a 12-h light/dark cycle, and had *ad libitum* access to water and food. After 2 days of acclimatization, oxygen consumption (VO_2_), carbon dioxide production (VCO_2_), and daily energy expenditure (heat) were monitored for five consecutive days using the Promethion Animal Energy Metabolism Monitoring System (Sable Systems International, Las Vegas, NV, USA).

### 2.6 HE staining

Adipose tissue specimens (the adipose tissue used in this study comprised epididymal white adipose tissue (eWAT) and inguinal white adipose tissue (iWAT)) was fixed in 4% neutral buffered paraformaldehyde (Bioshap, China, BL539A) for 24 hours, followed by graded dehydration, paraffin embedding, and sectioning into 4μm slices. Sections were deparaffinized, stained with haematoxylin for 5–10 minutes, counterstained with eosin for 1–2 minutes, washed twice with 70% ethanol. The sections were then observed under an inverted microscope (Leica, Germany) and images were captured.

### 2.7 Analysis of triglyceride and total cholesterol levels

Venous blood was collected from mice and centrifuged at 3000 rpm for 15 minutes in a refrigerated centrifuge at 4°C. The supernatant serum was collected and analysed for total cholesterol (T-CHO) and triglyceride (TG) content using a total cholesterol and triglyceride assay kit (Jiancheng, Nanjing, China).

### 2.8 Measurement of malondialdehyde levels

Malondialdehyde (MDA) levels were measured using an MDA assay kit (Jiancheng, Nanjing, China), normalised to total protein determined by BCA reagent (Thermo Corporation, Rockford, IL, USA). Results were ultimately expressed in mgprot/mL.

### 2.9 Inflammatory factors and cyclic GMP-AMP assay

Levels of inflammatory cytokines CCL4, TNF-α, IL-16, and IL-1β, and adipose tissue cGAMP levels, were measured using an ELISA kit (Jianglai, Shanghai, China). Protein content was determined using BCA reagent (Thermo Corporation, Rockford, IL, USA) for subsequent normalisation, with final results expressed in pg/mg.

### 2.10 Evaluation of mtDNA copy number

Adipose tissue was homogenized and centrifuged, and total DNA was extracted using the QIAamp DNA Mini Kit (Qiagen, Hilden, Germany). mtDNA copy number was quantified by real-time PCR using mitochondrial D-loop and GAPDH primers. The primer sequences are listed in **Supplementary Table S1**. The mtDNA copy number was assessed by the ratio of mtDNA to nuclear DNA and further analyzed using the 2^-ΔΔCt^ method.

### 2.11 Adipose tissue cell flow cytometry

Mouse epididymal white adipose tissue was digested with collagenase and processed into single-cell suspensions. In the same reaction tube, directly add the pre-titrated concentration of the fluorescent antibody mixture:F4/80-PE(BioLegend, 111703)、CD45-APC/CY7(BioLegend, 157204)、CD11B-PCP5.5(BioLegend, 101227)、CD86-APC-R700(BioLegend, 105023)和CD206-APC(BioLegend, 141708). All sample tubes were analysed individually on a flow cytometer (Beckman Coulter, Indianapolis, IN, USA).The sorting strategy is as follows: first exclude fragmented cells, then delineate immunological cell populations based on forward scatter (FSC-A) and side scatter (SSC-A) parameters. Subsequently, the CD45+ white cell population is sorted. Within the CD45+ cell population, all macrophage populations are identified by double-positive expression of CD11b and F4/80. M1 macrophages are defined as CD86+ cells within the total macrophage gate, whilst M2 macrophages are defined as CD206+ cells within the same gate. Finally, data analysis was performed using FlowJo software.

### 2.12 RNA sequencing

RNA sequencing (RNA-Seq) was performed by Majorbio (Shanghai, China), and transcriptome libraries were prepared using 1 μg of total RNA with a TruSeq RNA Sample Preparation Kit from Illumina (San Diego, CA, USA). Thereafter, the 300-bp complementary DNA (cDNA) target fragment size was selected using 2% low-range super agarose, followed by 15 cycles of PCR amplification using the Phusion DNA polymerase system. Following quantification using TBS380, bipartite RNA-seq (https://github.com/OpenGene/fastp) and clean reads were individually aligned to reference genomes with targeted patterns using HISAT2 (https://daehwankimlab.github.io/hisat2) software. RSEM (http://deweylab.biostat.wisc.edu/rsem/) was used to quantify the gene. Finally, differential expression analysis was performed using DESeq2 (http://bioconductor.org/packages/stats/bioc/DESeq2/).

### 2.13 Reverse transcriptase-quantitative PCR

Total RNA was extracted using TRIzol (Invitrogen, USA), and the RNA concentration in each sample was determined. A reverse transcriptase (RT)-PCR kit (TaKaRa, Seta Tsugaru-cho, Shiga, Japan) was used to reverse transcribe cDNA, and SYBR Green PCR Premix (QIAGEN, Schilden, Germany) was used for quantitative PCR (qPCR) analysis. Amplification curves were monitored using GeneExplorer (BORI, Hangzhou, China). The qPCR primers were synthesized by Shanghai Bioengineering (Sangon, Shanghai, China), and the primer sequences are listed in **Supplementary Table S1**. Finally, the mRNA results were analyzed using the 2^-ΔΔCt^ method, with *Gapdh* as the housekeeping gene.

### 2.14 Western blotting analysis

Mouse adipose tissue was homogenised and lysed in RIPA buffer (Thermo Fisher Scientific, USA) containing protease inhibitor (Solaibao, China). The lysate was subsequently subjected to Western blot analysis. Proteins were separated using sodium dodecyl-sulfate polyacrylamide gel electrophoresis and transferred to PVDF membranes. After blocking with 5% milk at room temperature for 1 h, the membranes were incubated overnight at 4 °C with the following primary antibodies: anti-tubulin (Proteintech, 11224-1-AP), anti-NRF2 (Proteintech, 14396-1-AP), anti-STING (CST (CST, 13647S), anti-pTBK1 (CST, 5483S), anti-TBK1 (CST, 3504S), anti-pIRF3 (CST, 4947S), and anti-IRF3 (Proteintech, 11312-1-AP), followed by incubation with the secondary antibody, anti-rabbit (Zhongshan Jinqiao, ZB-2301). Perform grey-scale analysis of the target bands using ImageJ software and conduct relative quantitative analysis of the target protein using α-tubulin as an internal reference.

### 2.15 Data analysis

Statistical analysis and graphing were performed using GraphPad Prism 10 software. Quantitative data are expressed as mean ± standard deviation. The experimental design comprised two factors: ‘genotype’ and ‘diet’. Therefore, two-way analysis of variance was employed to assess their main effects and interactions. For pairwise comparisons under specific conditions, unpaired t-tests were used. All hypothesis tests were conducted with P < 0.05 as the threshold for statistical significance.

## 3 Results

### 3.1 Establishment of an adipose tissue-specific *Nrf2* knockout mouse model

**Figure 1:**
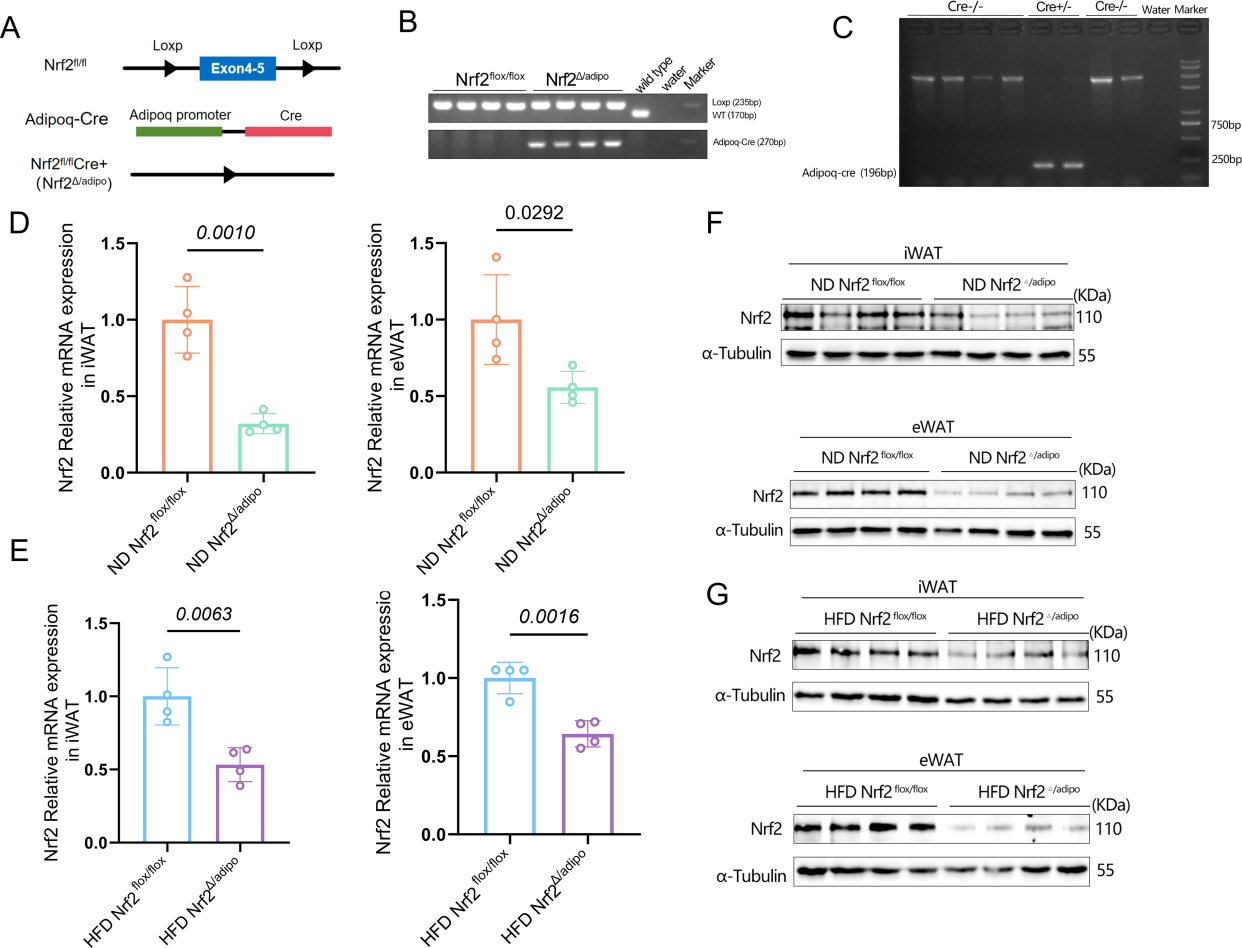
Establishment of an Adipose Tissue-Specific *Nrf2* Knockout Mouse Model. **(A)** Knockout strategy for adipocyte-specific *Nrf2* knockout mice, with *LoxP* sites (represented as triangles) flanking exons 4–5 of *Nrf2*. **(B)** Genomic DNA from mouse toes was analyzed via polymerase chain reaction (PCR) using *Loxp1, Loxp2*, and *Adipoq*-Cre-specific primers. **(C)** Identification of genomic DNA from mouse adipose tissue. **(D)** RT-qPCR analysis of *Nrf2* mRNA expression in iWAT and eWAT from *Nrf2^flox/flox^* and *Nrf2^△/adipo^* mice fed ND (n = 4). **(E)** RT-qPCR analysis of Nrf2 mRNA expression in iWAT and eWAT from *Nrf2^flox/flox^* and *Nrf2^△/adipo^* mice fed a HFD (n=4). **(F)** WB detection of Nrf2 protein expression levels in iWAT and eWAT from *Nrf2^flox/flox^* and *Nrf2^△/adipo^* mice under ND (n=4). **(G)** WB detection of Nrf2 protein expression levels in iWAT and eWAT from *Nrf2^flox/flox^* and *Nrf2^△/adipo^* mice fed a HFD (n=4). All data were obtained from at least two independent experiments. Statistical significance for panels **(D, E)** was determined using a two-tailed Student’s t-test, and data are expressed as mean ± SEM. Exact P-values are indicated in the respective figures.

To investigate the specific function of NRF2 in adipose tissue, adipose tissue-specific *Nrf2* knockout mice (*Nrf2^Δ/adipo^*) were constructed by crossing *Nrf2^flox/flox^* mice with transgenic mice carrying adipose tissue-specific Adipoq-Cre. Mouse toe and adipose tissues were PCR-amplified. Genotyping was performed (**Figures 1A–C**) to verify the accuracy of the model construction. The mRNA and protein expression levels of NRF2 in adipose tissue were further analyzed using real-time fluorescence qPCR and western blotting, respectively (**Figures 1D–G**). Significantly lower Nrf2 expression was observed in the adipose tissues of *Nrf2^Δ/adipo^* mice than in *Nrf2^flox/flox^* mice, indicating the successful construction of the knockout model.

### 3.2 Adipose tissue-specific knockout of *Nrf2* alleviates high-fat diet-induced obesity

**Figure 2:**
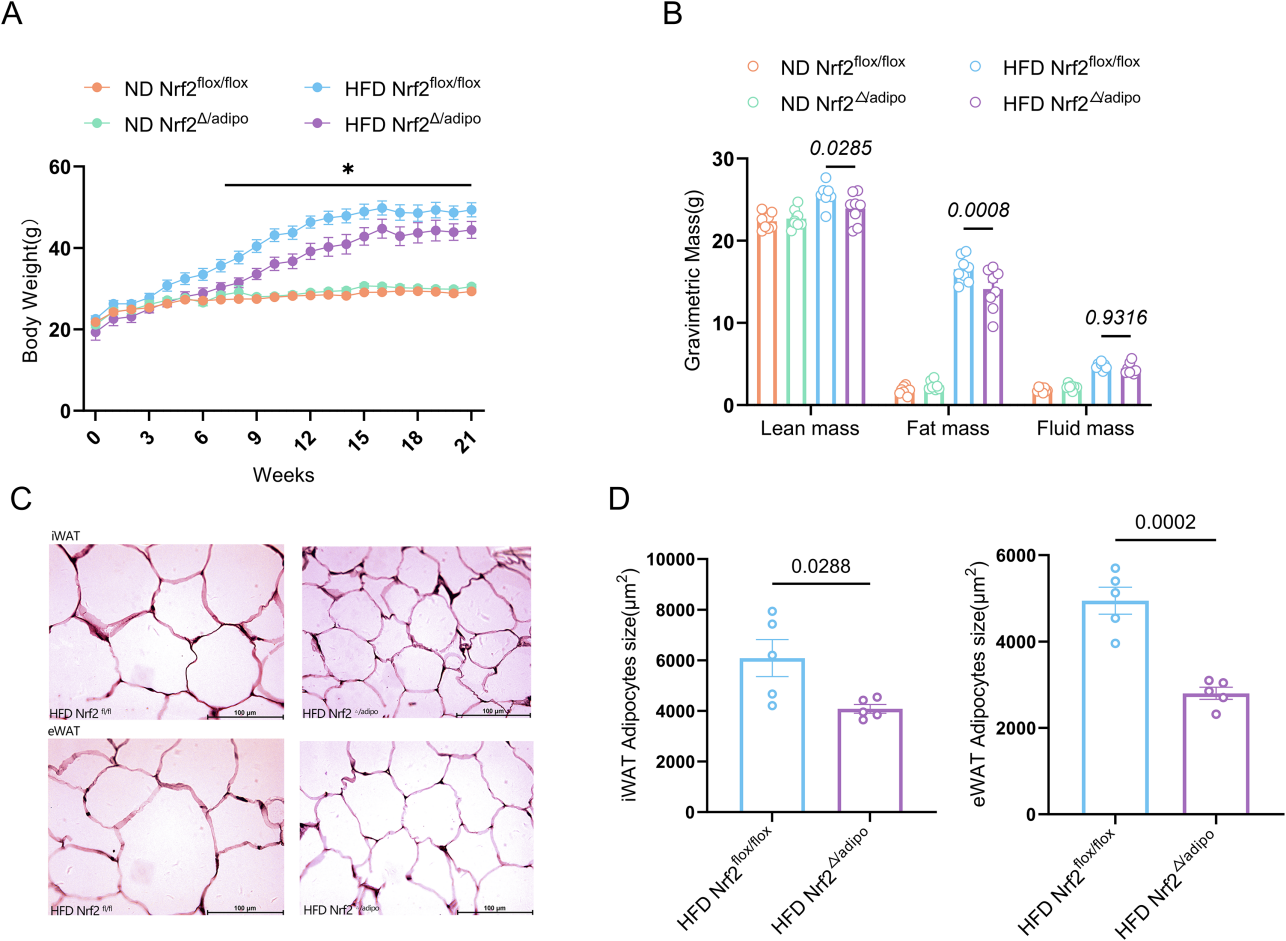
Effects of a HFD on body weight and body fat in *Nrf2^flox/flox^* and *Nrf2^△/adipo^* mice. **(A)** Body weight changes in *Nrf2^flox/flox^* and *Nrf2^△/adipo^* mice fed ND and HFD from week 0 to week 21 (n=8). **(B)** Body fat analysis in *Nrf2^flox/flox^* and *Nrf2^△/adipo^* mice under ND and HFD (n=8). **(C, D)** HE staining (×400) and cell area quantification analysis of iWAT and eWAT in *Nrf2^flox/flox^* and *Nrf2^△/adipo^* mice fed HFD (n=5).All data were derived from at least two independent experiments. Analysis of A-B employed a two-way ANOVA, whereas C-D was analysed with a two-tailed Student’s t-test. Data are presented as mean ± SEM, with exact p-values shown in the figures.

To assess the potential impact of adipocyte-specific *Nrf2* knockout on mouse obesity, we monitored the metabolic phenotypes of *Nrf2^flox/flox^* and wild-type mice after 21 weeks on either ND or HFD. At the commencement of feeding, no statistically significant differences in initial body weight were observed across all mouse groups (**Supplementary Figure 1**), thereby ensuring that the subsequent phenotypic variations observed were indeed attributable to dietary and genetic interventions rather than variations in initial body weight.Results showed no significant differences in body weight or body fat content among groups under ND. However, after 21 weeks on a HFD, *Nrf2^△/adipo^* mice exhibited slower weight gain and reduced body fat content compared to *Nrf2^flox/flox^* mice (**Figures 2A, B**). Further examination of adipocyte size in visceral (iWAT) and peripheral (eWAT) fat tissues via HE staining revealed that adipose tissue-specific *Nrf2* knockout significantly reduced adipocyte area in both iWAT and eWAT (**Figures 2C, D**). These findings indicate that adipose tissue-specific *Nrf2* knockout markedly attenuates HFD-induced obesity.

### 3.3 Adipose tissue-specific *Nrf2* knockdown attenuates HFD-induced insulin resistance

**Figure 3:**
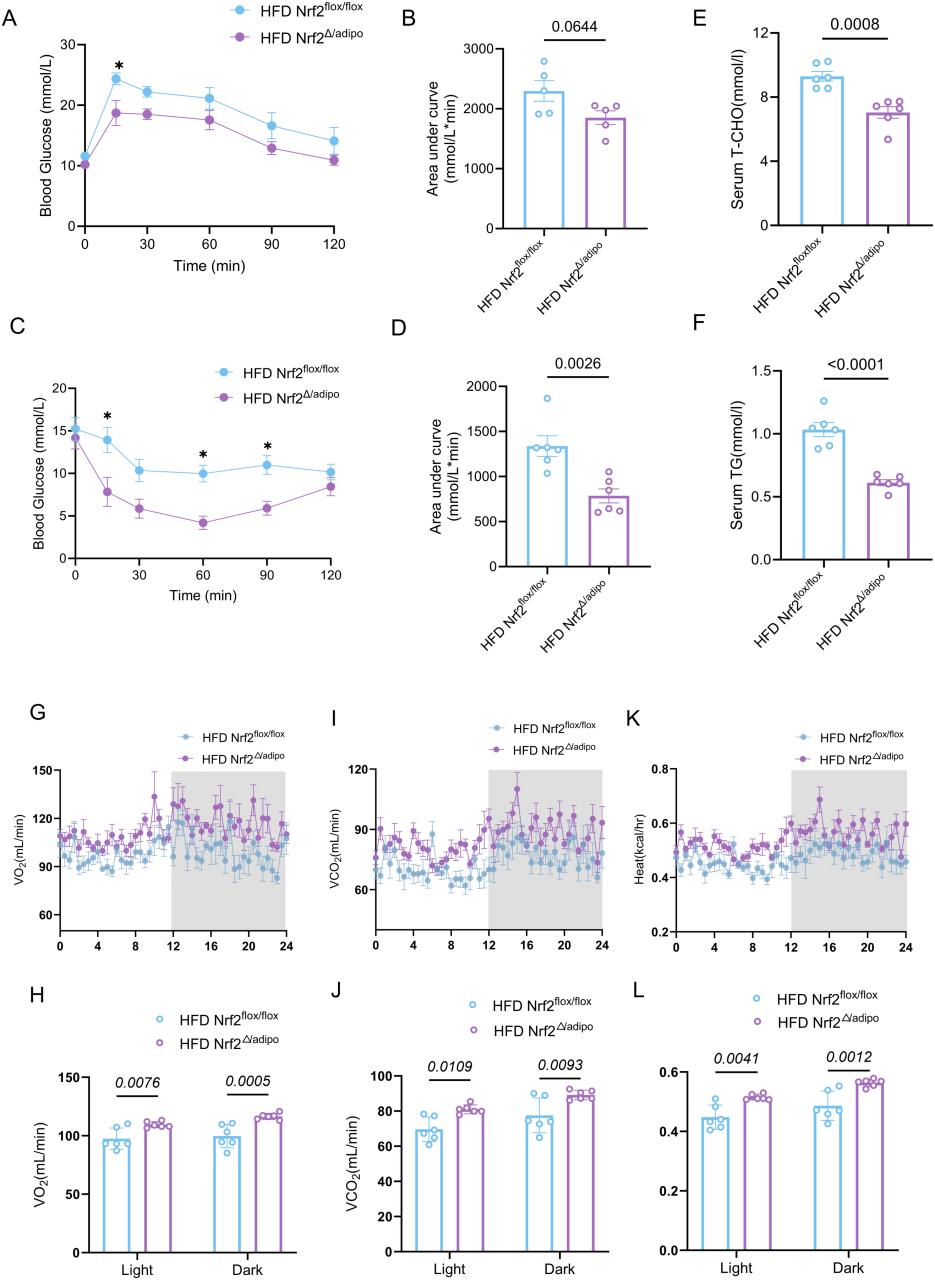
*Nrf2^Δ/adipo^* mice exhibited improved glucose tolerance, lower serum triglyceride (TG), total cholesterol (TC) levels, and higher energy expenditure (heat) than *Nrf2^flox/flox^* mice fed HFD. **(A, B)** Glycaemic changes and area under the curve in the intraperitoneal glucose tolerance test of *Nrf2^flox/flox^* and *Nrf2^Δ/adipo^* mice fed HFD (n =5). **(C, D)** Glycemic changes and area under the curve in the intraperitoneal insulin tolerance test of *Nrf2^flox/flox^* and *Nrf2^Δ/adipo^* mice on HFD (n = 6). **(E, F)** Serum TC and TG levels in *Nrf2^flox/flox^* and *Nrf2^Δ/adipo^* mice on HFD (n = 6). **(G–L)** Metabolic cage analysis of oxygen consumption (VO_2_), carbon dioxide production (VCO_2_), and heat in *Nrf2^flox/flox^* and *Nrf2^Δ/adipo^* mice on HFD (n = 6).All data were obtained from at least two independent experiments. Two-way analysis of variance was employed for **(A, C, H–L)**, while two-tailed Student’s t-tests were used for **(B, D, E, F)**. Data are presented as mean ± SEM, with precise p-values indicated in the figures.

Considering that obesity is closely associated with impaired glucose tolerance and insulin resistance (15), we systematically evaluated the glucose homeostasis of *Nrf2^flox/flox^* and *Nrf2^Δ/adipo^* mice. *Nrf2^Δ/adipo^* mice exhibited enhanced glucose and insulin tolerance after HFD feeding (**Figures 3A–D**). In addition, TG and TC levels were significantly downregulated in *Nrf2^Δ/adipo^* mice compared with those in the control group (**Figures 3E, F**), indicating the amelioration of lipid metabolism disorders.

To further assess the effect of adipose tissue-specific *Nrf2* knockdown on the basic metabolic activities of mice, the basal metabolic rate was monitored using a metabolic cage. VO_2_, VCO_2_, and heat levels were significantly increased in HFD *Nrf2^Δ/adipo^* mice compared with those in the control group (**Figures 3G–L**).

### 3.4 Nrf2 knockdown in adipose tissue inhibits the cGAS-STING signalling pathway in white adipose tissues

**Figure 4:**
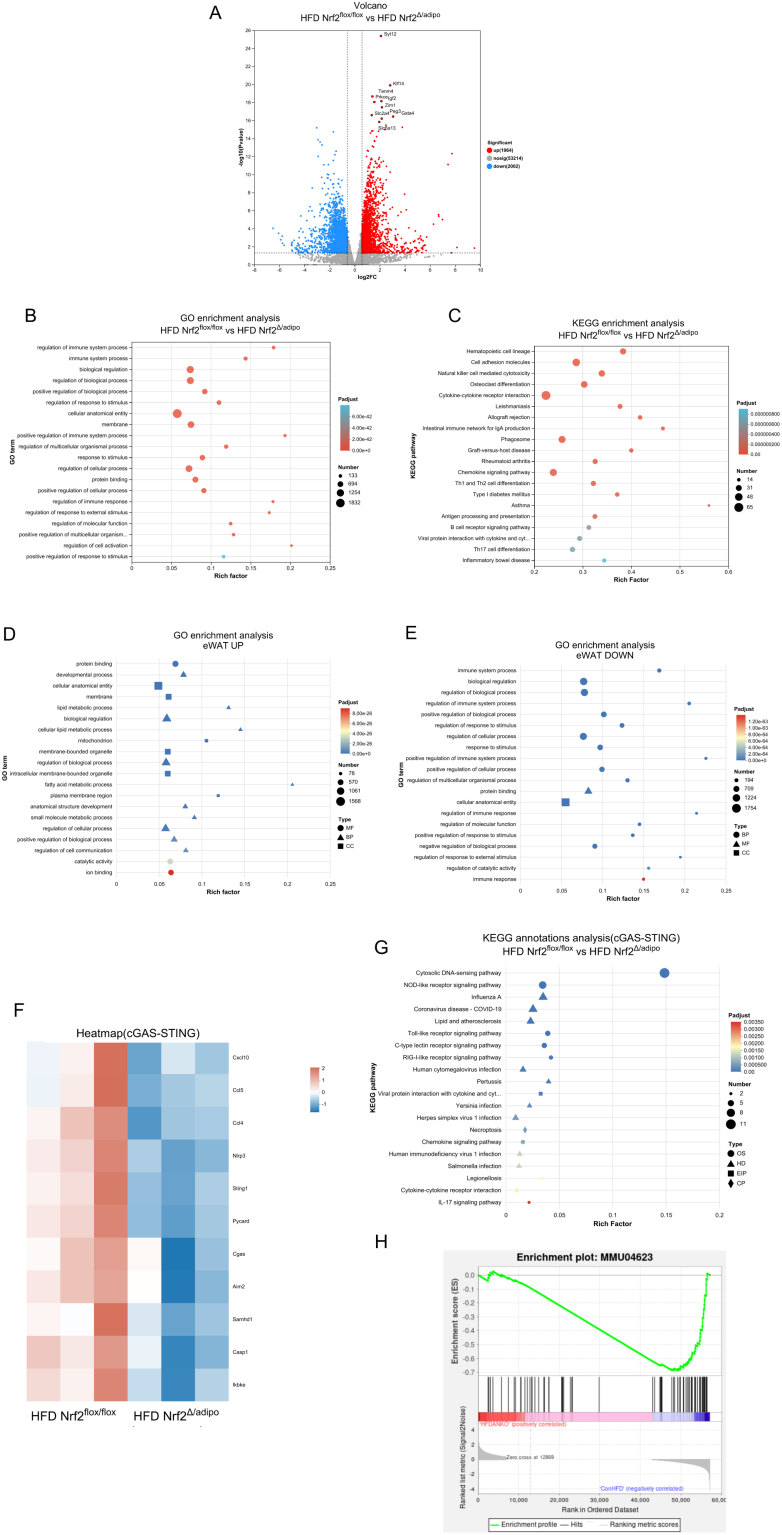
Transcriptome sequencing analysis of the effects of adipose tissue-specific *Nrf2* knockdown on signalling pathways in the eWAT of *Nrf2^flox/flox^* and *Nrf2^Δ/adipo^* mice on HFD. **(A)** Volcano plot of differentially expressed genes. **(B, C)** GO and KEGG enrichment analysis of differentially expressed genes, where dot size indicates the number of differentially expressed genes within each signalling pathway. **(D)** GO enrichment analysis of differentially expressed genes with increased expression. **(E)** GO enrichment analysis of downregulated differentially expressed genes. **(F)** Heatmap of differential gene expression levels associated with the cGAS-STING pathway. **(G)** KEGG enrichment analysis of differentially expressed genes associated with the cGAS-STING pathway. **(H)** GSEA enrichment analysis of the effects of adipose tissue-specific *Nrf2* knockout on cytoplasmic DNA-sensing signalling pathways.

To investigate the molecular mechanisms underlying improved obesity and insulin resistance observed after adipose tissue-specific *Nrf2* knockout, we performed RNA sequencing (RNA-seq) on eWAT from *Nrf2^flox/flox^* and *Nrf2^△/adipo^* mice fed a HFD. Differential expression analysis identified 3,966 differentially expressed genes (DEGs), comprising 1,964 significantly upregulated genes and 2,002 significantly downregulated genes (**Figure 4A**). Gene Ontology (GO) enrichment analysis and KEGG pathway enrichment analysis revealed significant enrichment in pathways such as ‘immunological response regulation’ and ‘lipid metabolism processes’ (**Figures 4B, C**), suggesting that *Nrf2* deficiency may simultaneously affect immune inflammation and metabolic homeostasis. Further analysis of up- and downregulated genes revealed that *Nrf2* deletion, broadly increased gene expression involved in fatty acid metabolism, lipolysis, and mitochondrial function, while significantly reducing gene expression linked to inflammatory responses and immune cell activation (**Figures 4D, E**) (**Supplementary Table 2**). This coordinated shift in metabolic and inflammatory gene expression prompted us to examine key signalling axes that could mediate such global transcriptional changes. During systematic evaluation of candidate pathways implicated in metabolic inflammation, the cGAS-STING pathway—serving as an essential link between cytoplasmic DNA sensing and innate immune activation—emerged as a key candidate. This pathway is known to be activated under conditions of obesity because mitochondrial DNA leaks into the cytoplasm, which drives chronic inflammation and insulin resistance.

Subsequently, we performed differential gene heatmap analysis targeting the cGAS-STING pathway (the core pathway mediating cytoplasmic DNA recognition). The results revealed that, compared to the control group, the expression levels of key components within this pathway—Ccl4, Ccl5, Cxcl10, Sting, Cgas, Casp1, and Trex1—were significantly downregulated (**Figure 4F**). To further validate the biological relevance of this pathway, KEGG enrichment analysis evaluated the clustering effects of differentially expressed genes at the pathway level. Results indicated that *Nrf2* deficiency significantly suppressed the ‘cytoplasmic DNA recognition pathway’ (**Figure 4G**). Subsequent GSEA enrichment analysis also downregulated trend in this pathway (**Figure 4H**). These findings suggest that adipose tissue-specific *Nrf2* knockout may alleviate obesity-associated inflammation and metabolic disorders by inhibiting the cGAS-STING pathway.

### 3.5 Adipose tissue-specific Nrf2 knockdown significantly inhibits activation of the cGAS-STING pathway

**Figure 5:**
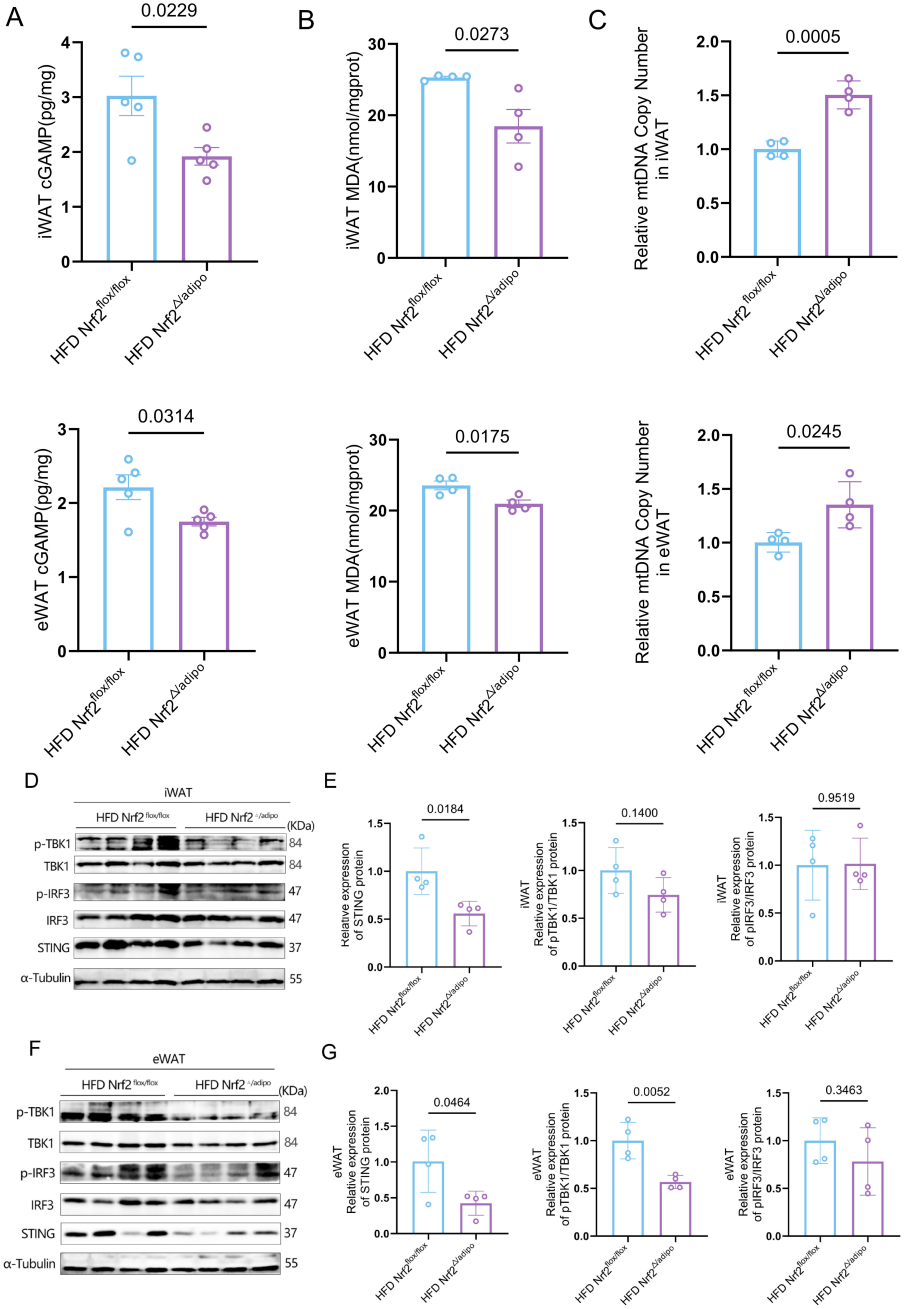
Adipose tissue-specific *Nrf2* knockdown improves lipid peroxidation and mitochondrial function and inhibits activation of the cGAS-STING pathway. **(A)** cGAMP levels in iWAT and eWAT (n = 5). **(B)** MDA levels in iWAT and eWAT (n = 4). **(C)** The relative copy number of total mitochondria in iWAT and eWAT (n=4). **(D, E)** STING, TBK1, p-TBK1, IRF3, and p-IRF3 protein expression levels in the iWAT of *Nrf2^flox/flox^* and *Nrf2^Δ/adipo^* mice fed a HFD (n = 4). **(F, G)** STING, TBK1, p-TBK1, IRF3, and p-IRF3 protein expression levels in the eWAT of *Nrf2^flox/flox^* and *Nrf2^Δ/adipo^* mice fed a HFD (n = 4). All data were derived from at least two independent experiments. The results above were analysed using a two-tailed Student’s t-test. Data are presented as mean ± SEM, with exact p-values indicated in the figures.

The cGAS-STING pathway is a central signalling mechanism for the intracellular detection of aberrant cytoplasmic double-stranded DNA (16). It is involved in inflammatory responses and metabolic regulation by activating type I interferons and proinflammatory factors. Oxidative stress in adipocytes during obesity induces mitochondrial membrane lipid peroxidation, leading to the leakage of mtDNA into the cytoplasm, which activates the cGAS-STING pathway (17, 18). To investigate whether Nrf2 deficiency in adipose tissue affects this pathway, we first assessed levels of its downstream messenger cGAMP. Results revealed significantly reduced cGAMP levels in the adipose tissue of *Nrf2^△/adipo^* mice fed a high-fat diet compared to controls (**Figure 5A**), indicating suppressed cGAS activity. To investigate the upstream mechanisms governing this pathway inhibition, we assessed mitochondrial status. Unlike typical oxidative stress models, lipid peroxidation markers (MDA) were significantly reduced in adipose tissue of *Nrf2^△/adipo^* mice (**Figure 5B**). MDA, the end product of lipid peroxidation, exhibited significantly reduced levels, indicating diminished lipid peroxidation and suggesting either lessened mitochondrial membrane damage or improved mitochondrial membrane integrity. Concurrently, real-time quantitative PCR analysis revealed a significant increase in the relative copy number of total mtDNA within the adipose tissue of *Nrf2^△/adipo^* mice (**Figure 5C**). Western blot analysis revealed markedly reduced STING protein levels within the cGAS-STING pathway in adipose tissue of *Nrf2^△/adipo^* mice fed a HFD, alongside decreased expression of p-TBK1 and p-IRF3 proteins (**Figures 5D–G**). This series of data indicates that Nrf2 deficiency may confine increased mtDNA within the organelle by improving mitochondrial health, thereby reducing its leakage into the cytoplasm and consequently suppressing the cGAS-STING pathway.

### 3.6 Adipose tissue-specific Nrf2 knockdown attenuates HFD-induced adipose tissue inflammation

**Figure 6:**
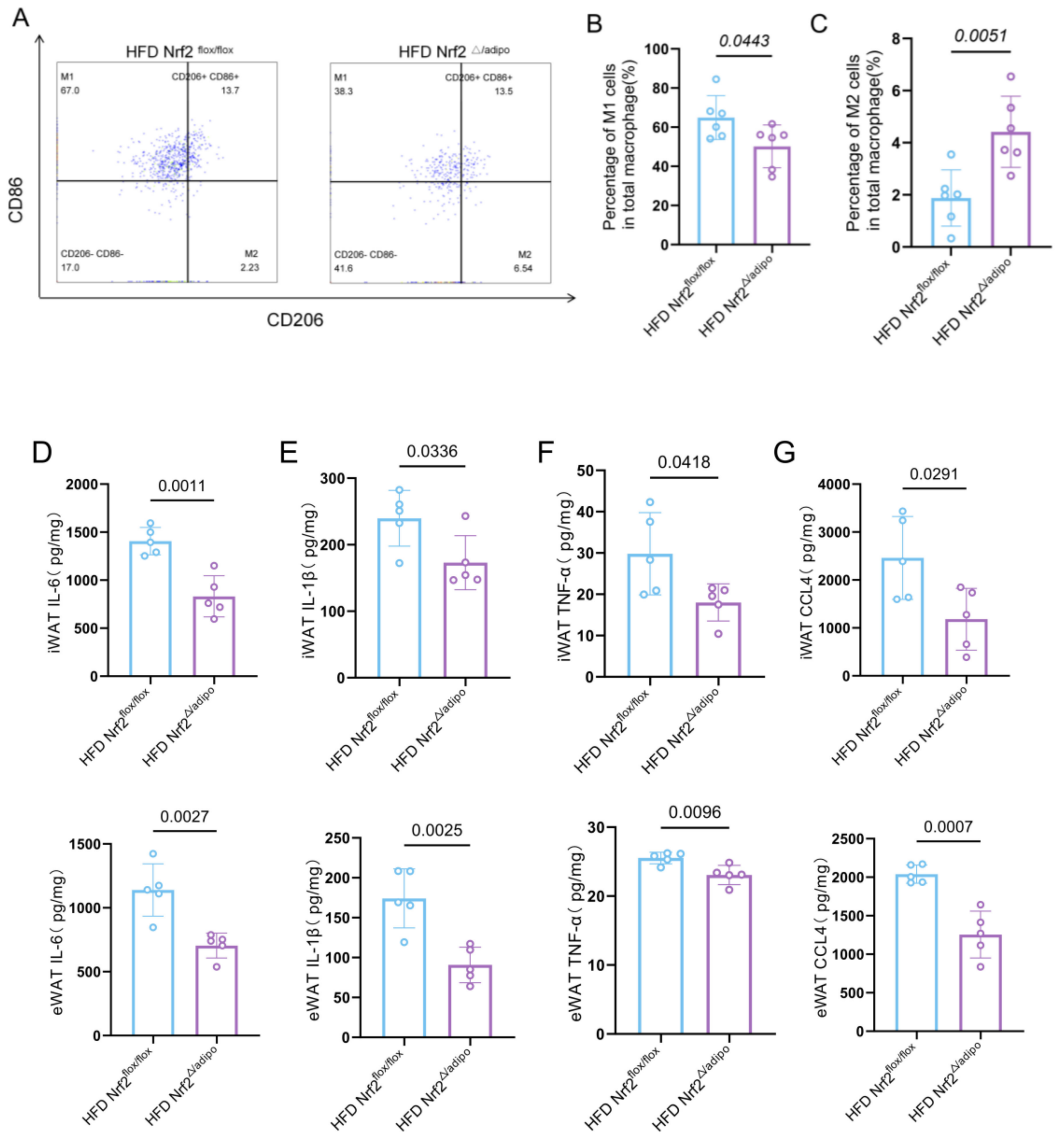
Reduced levels of inflammation in *Nrf2^Δ/adipo^* mice compared to those in HFD *Nrf2^flox/flox^* mice. **(A)** Flow cytometry analysis of M1 and M2 macrophage proportions in eWAT tissue from *Nrf2^flox/flox^* and *Nrf2^△/adipo^* mice fed a HFD. The gating strategy for flow cytometric analysis is shown in **Supplementary Figure 2**. **(B, C)** Statistical analysis of the proportion of M1 and M2 macrophages in eWAT tissue from *Nrf2^flox/flox^* and *Nrf2^△/adipo^* mice fed a HFD, by flow cytometry (n=6). **(D–G)** Levels of inflammatory cytokines IL-6, IL-1β, TNF-α and CCL4 in iWAT and eWAT tissues from *Nrf2^flox/flox^* and *Nrf2^△/adipo^* mice fed a HFD (n=5).All data were derived from at least two independent experiments. The results above were analysed using a two-tailed Student’s t-test. Data are presented as mean ± SEM, with precise p-values indicated in the figures.

Obesity is a chronic low-grade inflammatory state, primarily driven by adipose tissue inflammation (19). To investigate the effects of adipose tissue-specific Nrf2 knockdown on inflammatory phenotypes, we employed flow cytometry to analyse the distribution of macrophage subpopulations in the adipose tissue of *Nrf2^flox/flox^* mice and *Nrf2^Δ/adipo^* mice following HFD treatment (**Figure 6A**). Total macrophages were identified using CD11b and F4/80 surface markers, macrophage subpopulations were further distinguished using CD86 (M1-type macrophage marker) and CD206 (M2-type macrophage marker). Notably, the proportion of M1-type macrophages was significantly reduced, whereas that of M2-type macrophages was elevated in the adipose tissues of *Nrf2^Δ/adipo^* mice fed a HFD compared with those in *Nrf2^flox/flox^* mice (**Figures 6B, C**).

Levels of inflammatory factors in the adipose tissues of the mice were measured using ELISA. The proinflammatory factors IL-6, IL-1β, TNF-α, and CCL4 were downregulated in HFD *Nrf2^Δ/adipo^* mice compared with those in the control group (**Figures 6D–G**). Collectively, these results suggest that adipose tissue NRF2 deficiency significantly ameliorates adipose tissue inflammation by promoting macrophage polarization toward the M2 type and inhibiting proinflammatory factors.

## 4 Discussion

This study established an adipose tissue-specific Nrf2 knockout mouse model, and showed that the absence of Nrf2 in adipocytes significantly alleviates high-fat diet-induced obesity, insulin resistance, and adipose tissue inflammation. These effects are closely associated with inhibition of the cGAS-STING signalling pathway.

Nrf2, the core transcription factor of the cellular antioxidant defence system, has traditionally understood to mitigate oxidative stress by activating antioxidant enzymes such as HO-1 and NQO1 (20). However, adipose tissue-specific knockout of Nrf2 in this study, phenomena inconsistent with classical understanding were observed: adipose tissue-specific knockout of Nrf2 mitigated HFD-induced obesity and reduced adipose tissue inflammation. To further clarify this phenomenon, we also performed RNA-seq analysis of inflammation-related genes. This revealed that Nrf2 deficiency resulted in a significant downregulation of mRNA expression for multiple key pro-inflammatory factors, including Nlrp3, Tnfαip6, and Ccl5 (**Supplementary Figure 3A**). Although this finding contradicts the protective role of Nrf2 reported in most tissues, it is consistent with reports indicating that Nrf2 deficiency in adipose tissue reduces lipid accumulation in ob/ob mouse models (21–23). This suggests that Nrf2 function in adipose tissue may be regulated by leptin signalling or metabolism status. Moreover, interactions between Nrf2 and key adipogenic factors such as PPARγ may further contribute to functional heterogeneity across distinct fat depots (e.g., visceral versus subcutaneous fat) (24, 25). Overall, the biological effects of Nrf2 appear highly context dependent, shaped by tissue type and the organism’s metabolic state. In systemic Nrf2 knockout models, dysfunction in major metabolic organs such as the liver may dominate the overall phenotype. Conversely, in an obese context, Nrf2 in adipose tissue may engage with non-canonical pathways like cGAS–STING, influencing energy storage and inflammatory regulation that differ from—or even oppose—its classical antioxidant function. Therefore, tissue-specific regulation of Nrf2 may offer therapeutic value for metabolic disease therapeutics, although further systematic investigation remains essential.

Mechanistic studies indicate that the ameliorative effects of Nrf2 deficiency on metabolism and inflammation are not mediated through its classical antioxidant pathways. We first observed that Nrf2 knockout markedly reduced malondialdehyde (MDA) levels in adipose tissue (**Figure 5B**), suggesting a reduction in oxidative stress. This creates conditions conducive to maintaining mitochondrial membrane structural integrity (26).Subsequently, we observed an increase in the total mtDNA copy number within adipose tissue (**Figure 5C**). This suggests that the overall number of mitochondria may increase, potentially reflecting enhanced mitochondrial biosynthesis or quality control mechanisms. We hypothesise that structurally intact and functionally competent mitochondria may contribute to reducing the leakage of mtDNA into the cytoplasm. Although this study did not directly detect cytoplasmic mtDNA, this inference is indirectly supported by downstream cGAS-STING pathway analysis: levels of the cytoplasmic second messenger cGAMP were significantly reduced (**Figure 5A**), while phosphorylation signals within the STING-TBK1-IRF3 pathway were markedly suppressed (**Figures 5D–G**). To further investigate the potential basis for metabolic improvement, Through differential gene clustering and GSEA analysis, we identified activation of the AMPK signalling pathway (**Supplementary Figure 3B**). As a central regulator of cellular energy metabolism, AMPK pathway activation not only promotes substrate utilisation and energy production (27), but also enhances metabolic efficiency by optimising existing mitochondrial function (28, 29). This finding provides a potential molecular clue for explaining the increased energy expenditure and elevated metabolic rate observed in Nrf2-deficient mice in adipose tissue. In summary, our results support a proposed functional model: adipose tissue Nrf2 deficiency may alleviate obesity-associated inflammation by mitigating oxidative stress, improving mitochondrial function, and maintaining membrane integrity, thereby potentially reducing mtDNA leakage into the cytoplasm and ultimately suppressing the cGAS-STING pathway. This mechanism aligns with the ‘mtDNA-cGAS-STING axis drives obesity-associated inflammation’ model proposed by Bai et al. (30).

This study further showed that adipose tissue-specific knockout of Nrf2 significantly ameliorates HFD-induced systemic insulin resistance. Although the genetic manipulation was restricted to adipose tissue, the systemic metabolic improvements likely represent a multi-organ synergistic effect. We hypothesise that systemic insulin sensitivity may be enhanced through the following mechanisms: firstly, the alleviation of localised adipose tissue inflammation and reduced circulating pro-inflammatory factor levels may mitigate the inhibitory effects of chronic low-grade inflammation on insulin signalling (31, 32). Second, decreased circulating lipid levels (**Figures 2E, F**) may effectively alleviate lipid accumulation and lipotoxicity in peripheral organs such as the liver and skeletal muscle (33, 34). Furthermore, improved adipose tissue function may promote the secretion of beneficial adipokines such as adiponectin, remotely regulating insulin signalling in the liver and muscles via endocrine pathways (35–37). Collectively, these findings suggest that systemic improvements in insulin sensitivity may be closely linked to enhanced adipose tissue function, potentially involving cross-organ synergistic interactions across metabolic, inflammatory, and endocrine pathways.

Moreover, this study demonstrates that adipose tissue-specific knockout of Nrf2 increases energy expenditure in mice, as indicated by higher oxygen consumption, carbon dioxide production, and thermogenic capacity, together reflect an elevated basal metabolic rate. Because thyroid hormones act as primary regulators of resting energy expenditure, and their activity may be suppressed by chronic inflammatory states (38, 39), we hypothesise that the anti-inflammatory effects triggered by Nrf2 deficiency may indirectly enhance systemic energy expenditure by restoring thyroid hormone sensitivity. Future studies that combine animal models integrating Nrf2 deficiency with thyroid function intervention will facilitate precise delineation of the relative contributions of adipose tissue-autonomous effects versus systemic endocrine regulation in energy metabolism.

This study presents several noteworthy limitations. Firstly, Although our data support a model whereby Nrf2 deficiency alleviates obesity-associated inflammation by improving mitochondrial health and suppressing the cGAS-STING pathway, future studies must directly visualise and quantify mtDNA release. Employing a primary adipocyte culture system—which enables more efficient and controllable cytoplasmic fractionation—is crucial for confirming the presence of mtDNA in the cytoplasm and establishing its pivotal role as the primary driver of cGAS activation in this context. Secondly, our RNA-seq analysis results lack validation using independent methods such as qPCR. Although we employed stringent thresholds for differential gene screening, the absence of subsequent validation remains a technical shortcoming.

In summary, this study clarifies the mechanism by which Nrf2 in adipose tissue regulates obesity and associated metabolic inflammation via the non-canonical cGAS–STING pathway (Supplementary Figure 4). This not only provides a novel perspective on understanding the pivotal role of adipose tissue in metabolic diseases but also offers a potential new therapeutic target for obesity and related metabolic disorders. Future research should examine the functional heterogeneity of Nrf2 across different adipose tissue depots and its specific mechanisms in metabolic regulation.

## Data Availability

The datasets generated and/or analyzed during the current study are available from the corresponding author upon reasonable request.
