# Supplementary material for: Adipose tissue-specific Nrf2 knockdown inhibits the cGAS-STING pathway to attenuate inflammation in obese mice
Source: Front Endocrinol (Lausanne). 2026 Jan 12;16:1711793. doi: 10.3389/fendo.2025.1711793 (PMC12832232; doi:10.3389/fendo.2025.1711793)
Supplement: Supplementary file 1 [file DataSheet1.docx]

**Supplementary materials**

Gene primer sequences:

| Gene name | Primer sequence (5'→3'） |
| --- | --- |
| *FLOX-F* | GGCCTGTAAACTACAAGTCCAT |
| *FLOX-R* | CTAGCAGAATGCTTCAAACAACAG |
| *Adipoq-cre-F* | ACGGACAGAAGCATTTTCCA |
| *Adipoq-cre-R* | GGATGTGCCATGTGAGTCTG |
| *Nrf2-F* | CCAGAAGCCACACTGACAGAAATGG |
| *Nrf2-R* | AGTGGAGAGGATGCTGCTGAAAGAA |
| *Dloop -F* | AATCTACCATCCTCCGTGAAACC |
| *Dloop -R* | CAGTTTAGCTACCCCCAAGTTTAA |
| *Gapdh-*F | ACCTCAACTACATGGTCTACA |
| *Gapdh*-R | CTTCCCATTCTCGGCCTTG |

**Table.S1 Gene primer sequences**

Top 50 differentially expressed genes


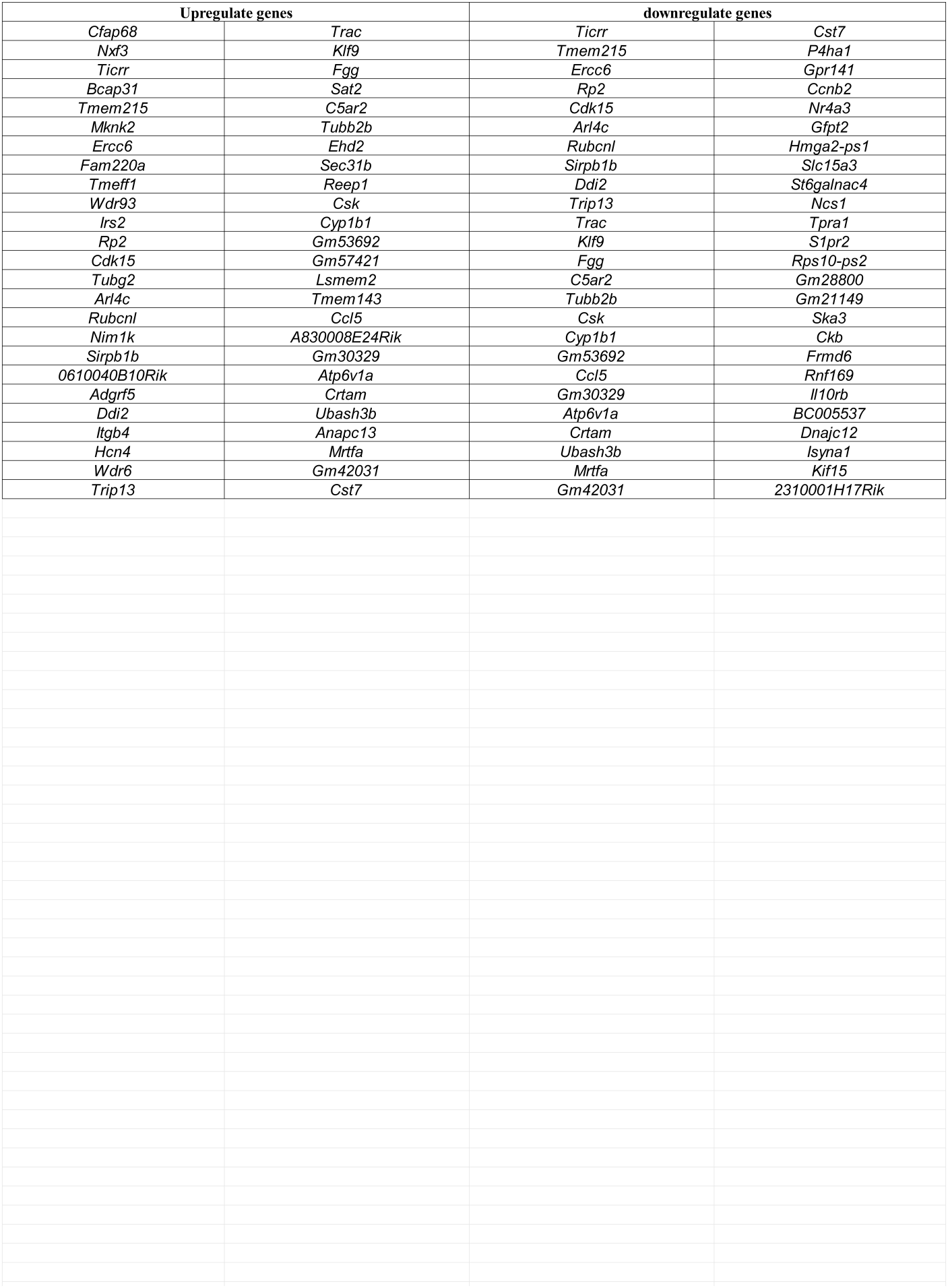
**Table.S2 List of differentially expressed genes**


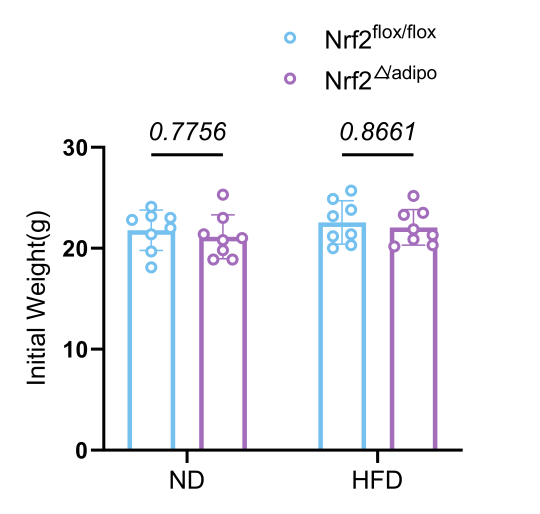


**Fig. S1** Initial body weight of *Nrf2^flox/flox^* mice and *Nrf2^△/adipo^* mice prior to normal diet (ND) and high-fat diet (HFD) (n=8).A two-way analysis of variance was employed. Data are presented as mean ± standard error of the mean (SEM), with exact p-values indicated in the figures.


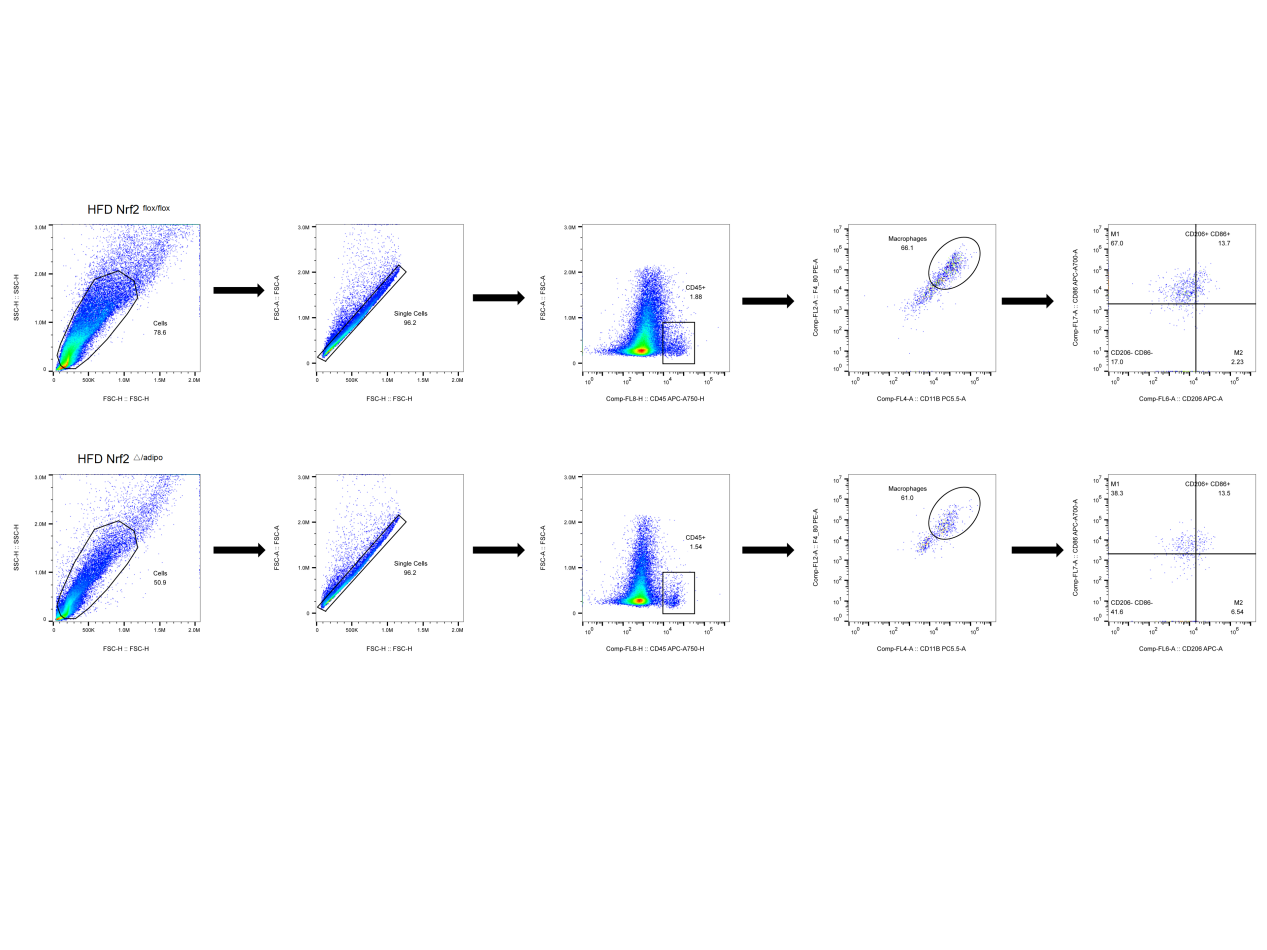


**Fig. S2 Gating strategy for identifying M1 and M2 macrophages in eWAT by flow cytometry.**Primary cell populations were defined based on forward scatter (FSC) and side scatter (SSC) characteristics, with debris excluded. leukocyte population were subsequently enriched by CD45 expression. Within this population, total macrophages were identified as double-positive for CD11b and F4/80, and further differentiated into M1 and M2 subtypes according to CD86 and CD206 expression levels.


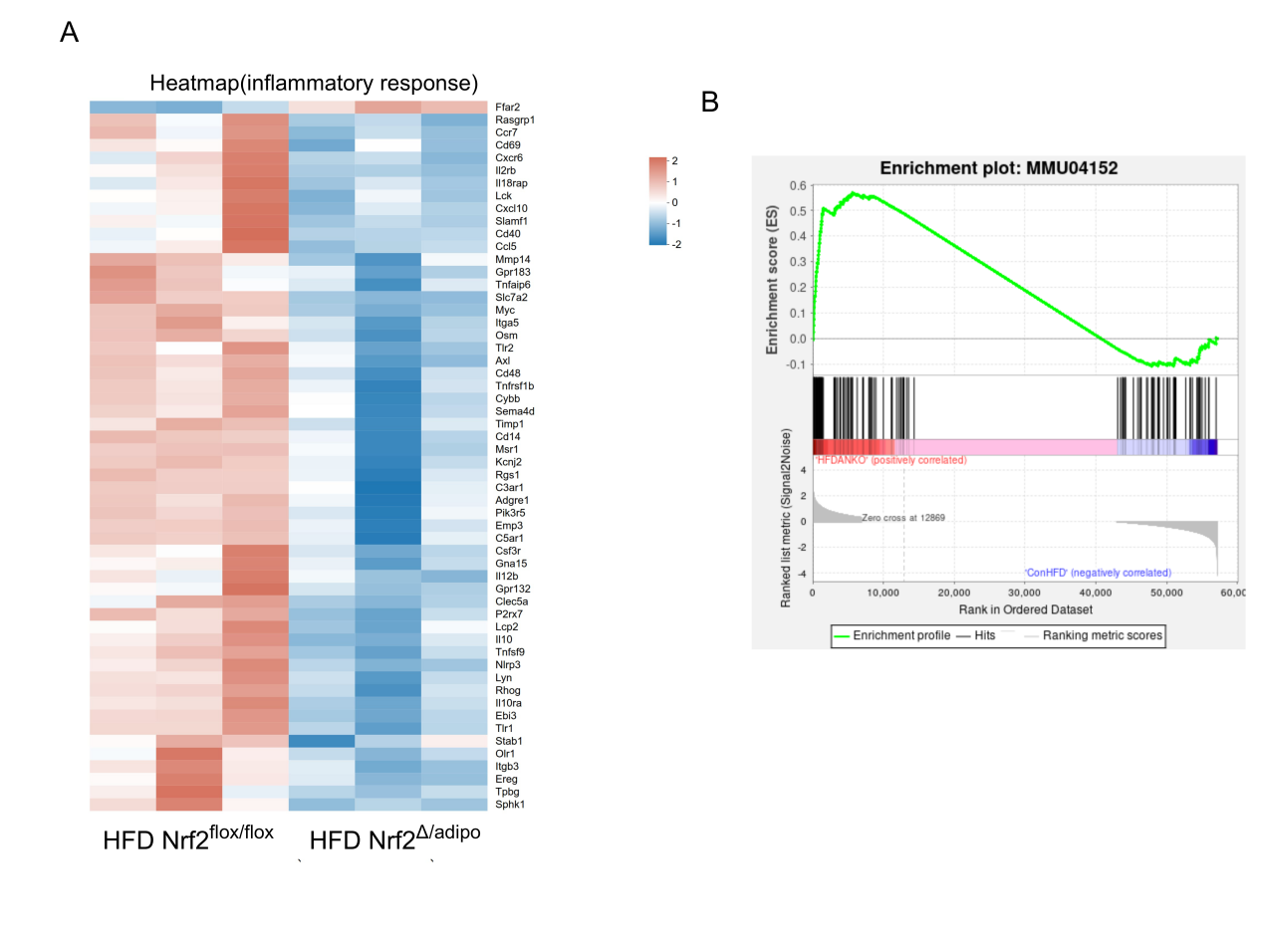


**Fig. S3** (A) Heatmap of Inflammatory Pathway-Related Genes.(B)GSEA enrichment analysis of the impact of Nrf2 knockout specific to adipose tissue on the AMPK signalling pathway.


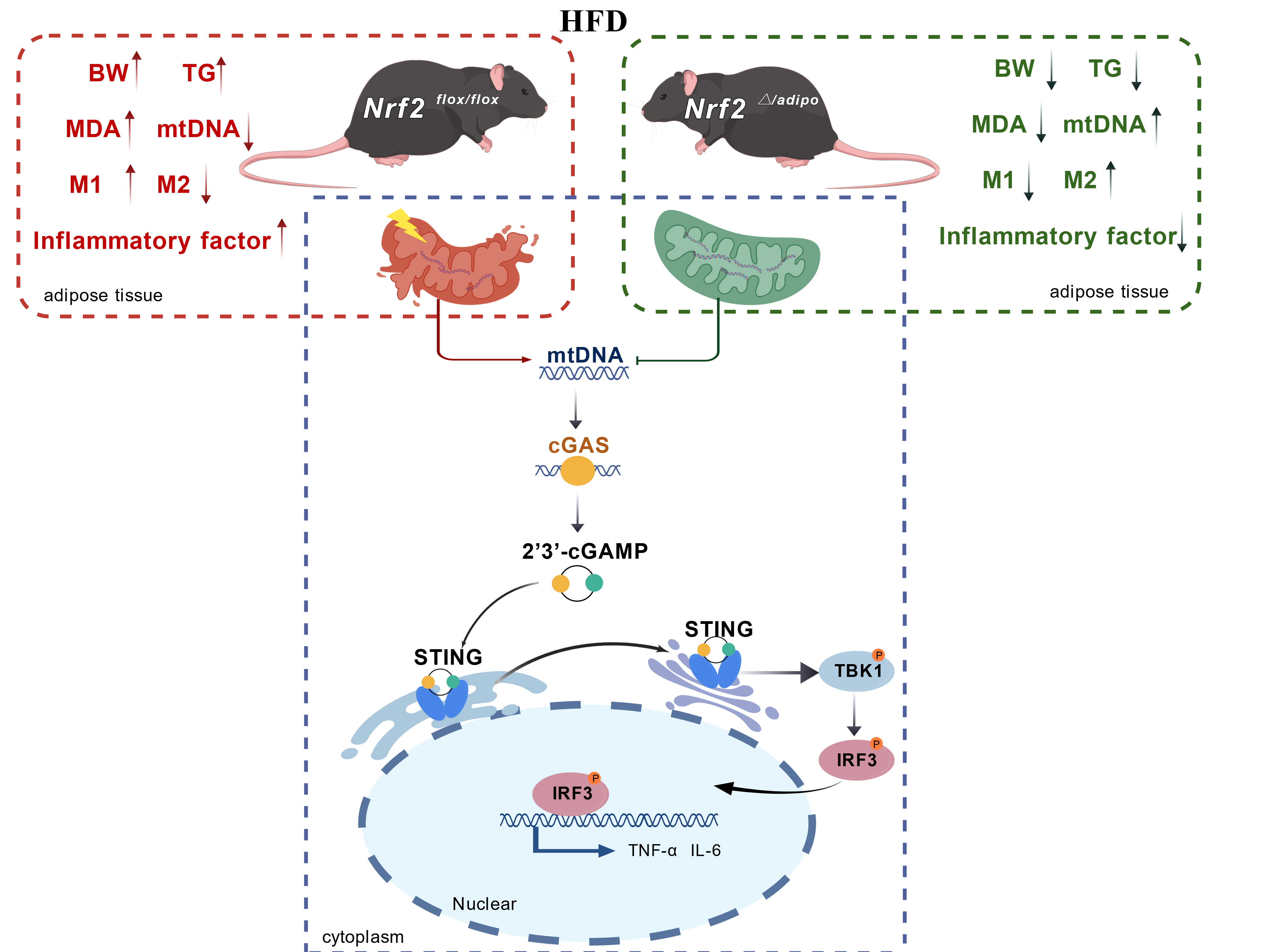


**Fig. S4** The schematic diagram demonstrates that adipose tissue-specific Nrf2 knockdown alleviates adipose tissue inflammation in obese mice by inhibiting the cGAS-STING pathway.
